# Superior Vena Cava as an Arrhythmogenic Substrate in Atrial Fibrillation: Anatomical, Electrophysiological and Clinical Perspectives

**DOI:** 10.3390/jcm14217456

**Published:** 2025-10-22

**Authors:** Szymon Zakrzewski, Maciej Wielusinski, Jaroslaw Kazmierczak, Lukasz Duda, Wiktoria Hoppe, Katarzyna Bazylewicz-Zakrzewska, Aleksandra Kladna, Radoslaw Marek Kiedrowicz

**Affiliations:** 1 Cardiology Department, Pomeranian Medical University, Powstancow Wlkp. 72, 70-111 Szczecin, Poland; 2 Department of Diagnostic Imaging and Interventional Radiology, Pomeranian Medical University, Unii Lubelskiej 1, 71-252 Szczecin, Poland; 3 Department of History of Medicine and Medical Ethics, Pomeranian Medical University, Rybacka 1, 70-204 Szczecin, Poland

**Keywords:** superior vena cava, atrial fibrillation, non-pulmonary vein triggers, catheter ablation

## Abstract

Catheter ablation is an established treatment method for atrial fibrillation. In most cases, the arrhythmia substrate is located within the pulmonary veins and their isolation has become the cornerstone of catheter ablation therapy. However, it is estimated that in 10–20% of patients, the trigger and/or sustaining factors for the arrhythmia are located outside the pulmonary veins. This is particularly relevant in patients with significant disease progression and persistent forms of AF, where these sites are likely responsible for treatment failure and arrhythmia recurrence. Identifying non-pulmonary vein triggers of AF and understanding their impact on disease progression is crucial but remains insufficiently explored. Gaining such insights offers hope for optimizing treatment strategies and improving outcomes for patients. The most commonly mentioned ectopic arrhythmogenic focus include the superior vena cava (SVC). Despite its clinical significance in arrhythmogenesis, the detailed electrophysiological properties of the SVC have received relatively limited research attention compared to other cardiac structures. Moreover, scientific papers that present extensive knowledge on SVC- from its embryology to the electrophysiology scarce. Therefore we would like to offer a comprehensive analysis of existing literature concerning SVC as an arrhythmogenic substrate for AF.

## 1. Introduction

Atrial fibrillation (AF) is the most common cardiac arrhythmia and is responsible for 20–30% of ischemic strokes. It may lead to dementia, heart failure and is an independent factor that can increase the risk of mortality by 3.5 times. Additionally, AF is often associated with the necessity for hospitalization; approximately 30% of patients require hospitalization at least once a year. The global number of individuals affected by AF has reached 45 million, with a lifetime risk of developing the condition affecting one in three individuals [1]. Catheter ablation is an established treatment method for atrial fibrillation [2]. In most cases, the arrhythmia substrate is located within the pulmonary veins (PV) [3] and their isolation has become the cornerstone of catheter ablation therapy [4]. However, it is estimated that in 10–20% of patients, the trigger and/or sustaining factors for the arrhythmia are located outside the pulmonary veins [5]. The most commonly mentioned ectopic arrhythmogenic foci include the superior vena cava (SVC), the posterior wall of the left atrium (LA), the left atrial appendage, crista terminalis, the coronary sinus and the vein of Marshall [6]. This is particularly relevant in patients with significant disease progression and persistent forms of AF, where these sites are likely responsible for treatment failure and arrhythmia recurrence [7]. Identifying the non-pulmonary vein triggers of AF and understanding their impact on disease progression is crucial but remains insufficiently explored. Gaining such insights offers hope for the optimization of treatment strategies and improvement of patient outcomes. Therefore, we would like to present a comprehensive analysis of existing literature concerning SVC as an arrhythmogenic substrate for AF.

## 2. Information Sources and Search Strategy

Four online databases: PubMed, Embase, Scopus, and Web of Science were searched on August 2025, ensuring the inclusion of both foundational studies and the most recent findings. The study selection criteria included randomized, non-randomized and observational clinical studies published in English, with no time restriction.

## 3. Superior Vena Cava Serving as the AF Arrhythmogenic Source

Among patients who exhibit non-PV triggers of atrial fibrillation SVC consistently represents a prominent source across multiple studies. In one of the earliest comprehensive studies examining non-PV triggers, Lin et al. documented SVC ectopy in 27 out of 73 non-PV foci (37.0%), making it the second most common non-PV trigger site after the left atrial posterior free wall among 240 patients with paroxysmal atrial fibrillation [8]. Recent large-scale clinical studies have provided more detailed epidemiological data on SVC-triggered atrial fibrillation prevalence. Takigawa et al. investigated a cohort of 865 PAF patients: SVC-associated triggers were present in 57 patients (6.6% of the whole cohort), while other non-PV, non-SVC foci were present in 68 (7.6%). This means that among all non-PV cases (N = 125), SVC triggers accounted for 45.6% of non-PV. There was further overlap: 14 out of the 68 non-PV, non-SVC patients also had SVC trigger episodes, indicating secondary multifocality, so the absolute figure for SVC involvement among non-PV sources could be even higher depending on classification. Thus, their data corroborate that, among non-PV foci, SVC represents a dominant proportion—that is roughly half [9].

Kawai et al. in their analysis of 1020 patients undergoing AF ablation procedures identified non-PV triggers in 10.6% of their total cohort. Within this subgroup SVC triggers constituted about 23% of all non-PV sources, ranking it among the top three non-PV trigger sites [10]. Miyata et al. reported that among non-PV-triggered AF, 40% originated from the SVC, the highest proportion among non-PV sites in their cohort, including cases with persistent AF induced by SVC firing (~10% of non-PV triggers) [11]. The highest reported prevalence of SVC-triggered atrial fibrillation was documented by Dong et al., who identified SVC triggers in 30 out of 130 patients (23.1%) with paroxysmal atrial fibrillation. Dong et al. found that in patients with non-PV triggers, SVC was responsible for about 69.8% of the non-PV ectopic foci, again confirming its major role as a non-PV arrhythmogenic source [12]. The higher detection rate in this study may be attributed to their aggressive protocol to elicit SVC triggers, as well as performing electrophysiological studies before circumferential pulmonary vein isolation, potentially avoiding the masking effects of autonomic changes following PV isolation. Overall, across studies focusing on patients with non-PV triggers, the prevalence of SVC-related AF triggers ranges roughly between 23% and 69.8% of all identified non-PV foci (Table 1).

This highlights the superior vena cava as a key non-pulmonary source necessitating targeted evaluation and, when indicated, ablation in this patient subset. However, it seems that the actual prevalence of truly SVC-dependent AF remains unknown. Atrial ectopy originating from SVC, either spontaneous or drug/pacing induced, does not necessarily translate into AF. It should be noted that differences in the reported proportions of SVC-related foci across studies may result from significant methodological variations and differences in patient inclusion criteria. Some analyses included only patients with paroxysmal AF, whereas others encompassed both paroxysmal and non-paroxysmal (persistent or long-standing persistent) forms. In addition, different approaches were used to report the frequency of SVC as an arrhythmogenic source; some studies expressed it as the proportion of SVC foci among all identified non-PV triggers, while others reported the percentage of patients or procedures in which such foci were detected. These differences in definitions and counting methods could substantially affect the reported percentages and the interpretation of the true prevalence and role of the SVC as an AF source. Moreover, the more aggressive induction protocol, the lower the specificity for non-PV trigger identification. On the other hand, AF induction due to SVC firing is hard to register and therefore a rarely observed phenomenon.

## 4. Induction Protocols Used for Non-PV Trigger Detection

Lin et al. employed a systematic stepwise algorithm previously described by Hsieh et al., 1999 [13], which represented one of the most comprehensive induction protocols. This five-step approach began with baseline observation for spontaneous AF, followed by an isoproterenol infusion of up to 4 μg/min, then high-dose adenosine bolus 18–24 mg. If these pharmacological methods failed, short-duration burst pacing (8 beats) was performed from the right atrium, coronary sinus and pulmonary veins under isoproterenol infusion, followed by high-current burst pacing from the right atrium and coronary sinus if needed. Finally, when pacing-induced AF was sustained for >5 min, external cardioversion (50–100 J) was performed with subsequent observation for spontaneous reinitiation patterns [8,13].

Kawai et al. implemented a pragmatic clinical approach using intravenous isoproterenol and adenosine triphosphate combined with repeated direct current cardioversion during AF sessions. This approach was applied across multiple ablation sessions, with notably higher detection rates in recurrent procedures (30.9%), compared to first sessions (6.3%) [10].

Yan Dong et al. used a systematic pre-pulmonary vein isolation (PVI) protocol in their randomized controlled trial. The standardized approach began with isoproterenol infusion (4 μg/min) to increase heart rate by more than 20% from baseline, followed by sequential rapid burst pacing (200 ms for 30 s) in coronary sinus, SVC, left superior PV and right superior PV electrodes. When initial methods failed, adenosine triphosphate boluses (40 mg) were administered during ventricular pacing (500 ms) to uncover ectopic beats initiating AF [12].

Takigawa et al. utilized a fundamentally different approach by performing induction only after extensive PVI completion. Their post-ablation protocol involved a sequential administration of isoproterenol (5–20 μg/min), followed by adenosine triphosphate (20–40 mg) to unmask dormant conduction, then rapid atrial pacing to induce AF. When pacing-induced AF was sustained, internal cardioversion was performed with observation for spontaneous reinitiation. This post-PVI timing resulted in a significantly lower SVC detection rate of 6.6% of the total patients, suggesting that prior PV ablation may mask non-PV trigger activity [9].

## 5. Anatomical and Histological Properties of the SVC

**Figure 1:**
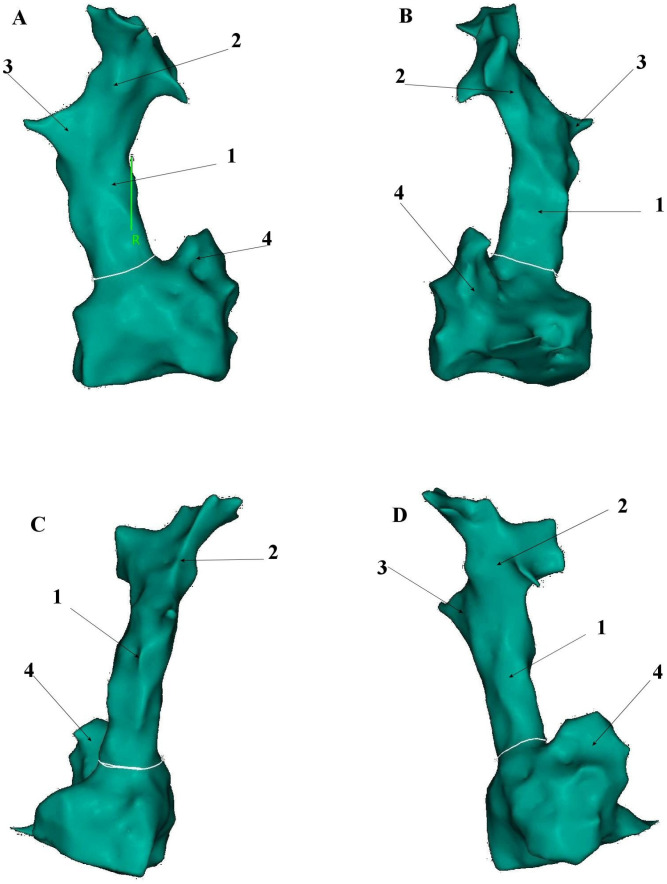
Presentation of superior vena cava anatomical relationship to the right atrium based on the electroanatomical shell created with CARTO^®^ 3 platform (Biosense-Webster, Diamond Bar, CA, USA). (**A**). Anteroposterior (AP) view; (**B**). Left anterior oblique (LAO) view; (**C**). Posteroanterior (PA) view; (**D**). Right anterior oblique (RAO) view. 1—Superior vena cava, 2—Left brachiocephalic vein, 3—Right brachiocephalic vein, 4—Right atrial appendage.

**Figure 2:**
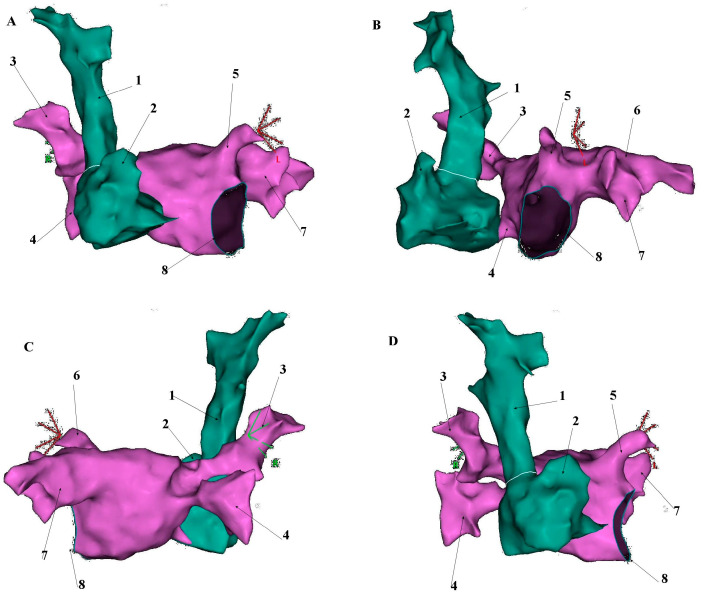
Presentation of superior vena cava anatomical relationship to the left atrium based on the electroanatomical shell created with CARTO^®^ 3 platform (Biosense-Webster, Diamond Bar, CA, USA). (**A**). Anteroposterior (AP) view; (**B**). Left anterior oblique (LAO) view; (**C**). Posteroanterior (PA) view; (**D**). Right anterior oblique (RAO) view. 1—Superior vena cava, 2—Right atrial appendage, 3—Right upper pulmonary vein, 4—Right lower pulmonary vein, 5—Left atrial appendage, 6—Left upper pulmonary vein, 7—Left lower pulmonary vein, 8—Mitral valve annulus.

**Figure 3:**
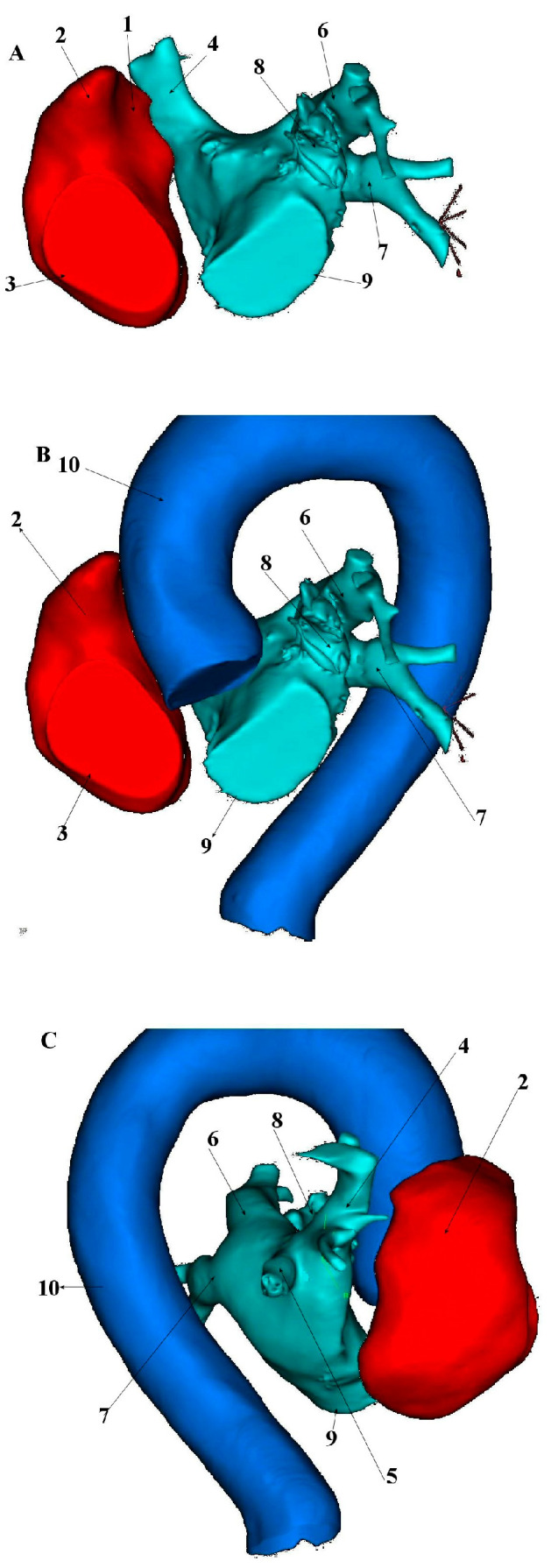
Presentation of superior vena cava anatomical relationship to the right and left atrium along with aorta, based on the computed tomography data incorporated into the CARTO^®^ 3 platform (Biosense-Webster, Diamond Bar, CA, USA). (**A**). Left anterior oblique (LAO) view without incorporated aorta; (**B**). Left anterior oblique (LAO) view with incorporated aorta; (**C**). Posteroanterior (PA) view. 1—Superior vena cava, 2—Right atrial appendage, 3—Tricuspid valve annulus, 4—Right upper pulmonary vein, 5—Right lower pulmonary vein, 6—Left upper pulmonary vein, 7—Left lower pulmonary vein, 8—Left atrial appendage, 9—Mitral valve annulus, 10—Aorta.

The superior vena cava develops embryologically from the right anterior cardinal vein, right common cardinal vein and the right horn of the sinus venosus during the 5th week of fetal development. Its average length in adults is 7.1 cm ± 1.4, with a maximum diameter of 2.1 cm ± 0.7 and exhibits an irregular cross-sectional shape on ECG-gated CT imaging [14,15]. The SVC courses through the right mediastinum, bordered laterally by the pleura and lung and medially by the trachea and ascending aorta. Major tributaries include the azygos vein, which arches over the right tracheobronchial angle to drain into the distal SVC [15] (Figure 1, Figure 2 and Figure 3).

The superior vena cava is characterized by the presence of cardiac muscle fibres that form so-called myocardial sleeves resembling similar structures in the pulmonary veins [16,17,18]. Histological and immunohistochemical studies revealed that myocardial sleeves in the SVC contain cardiomyocytes with phenotypes resembling Purkinje fibres and nodal cells. Moreover, strong desmin immunoreactivity has been observed in SVC myocardial sleeves, which is more abundant in conduction system cells than in working myocardium, suggesting a conduction-like phenotype for these cardiomyocytes [19]. These sleeves are predominantly located in the outer adventitial layer of the SVC wall [20]. In one autopsy study, where 620 human hearts were systematically examined, researchers found muscle sleeves in SVC in 78% of cases with a median of 4 mm maximal extension length [16]. More detailed examination at the histological level revealed that these fibres extend continuously from the atrial wall to the venous wall to a length of about 45 mm, reaching up to the level of the azygos vein. Cardiac myocytes in the SVC wall are 10–20 μm thick and form bundles arranged longitudinally, obliquely or circularly. They occupy one to two thirds of the vessel wall thickness, with their quantity decreasing towards the periphery. Cardiac muscle fibres are present in the SVC wall externally in relation to smooth muscle fibres. In the part closer to the heart, cardiac muscle fibres are numerous, while their quantity decreases towards the periphery. In the distal section at the level of the pericardium, longitudinally arranged cardiac cells are visible in the anterior wall but absent in the posterior wall [17].

Spach et al. (1972) examined the venae cava of human patients undergoing cardiac surgery and observed that electrical excitation waves spread from the atrium to 2–5 cm peripherally into the SVC, which corresponds to the range of cardiac muscle distribution observed in histological studies [21]. This suggests that the human SVC near the atrium actually contracts. Cardiac muscle fibres in the SVC extend continuously from the atrial wall containing the sinoatrial node. This means that the contraction of the venous cardiac muscles is synchronized with atrial contraction. Electrical excitation waves spread up and down the sinoatrial node in the lateral part of the SVC–atrium junction [17].

Myocardial sleeves are heterogeneous in their morphology, displaying complex arrangements that influence electrical conduction through this region [22]. Using canine models, Yeh and colleagues demonstrated significant differences in the assembly and spatial orientation of cardiomyocytes at the right atrium (RA), SVC and RA-SVC junction regions [23]. The heterogeneous cellular architecture at the SVC–RA transition creates a unique substrate that may support arrhythmia initiation and maintenance under certain conditions [19].

The presence of muscular sleeves in the SVC wall may contribute to its arrhythmogenic potential. The contraction of cardiac muscles in the SVC, which is synchronized with atrial contraction, may act as a functional valve preventing the backflow of blood from the atrium to the SVC during atrial contraction [17].

**Figure 4:**
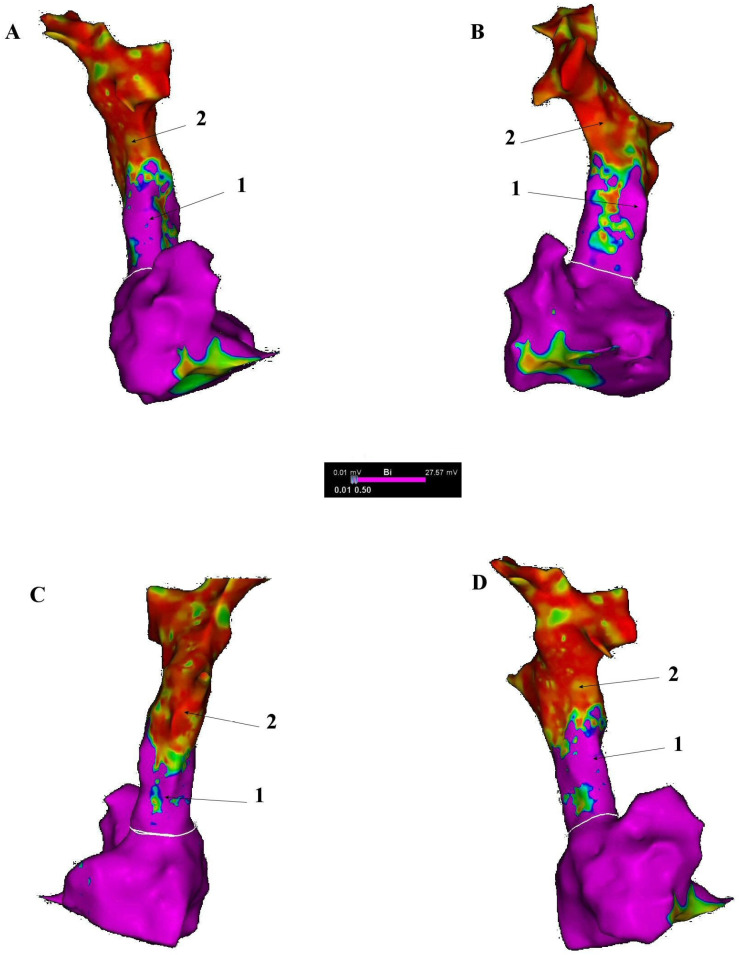
Superior vena cava myocardial sleeves detected on the basis of high-density, high-resolution voltage mapping performed with a Pentaray catheter, created with CARTO^®^ 3 electroanatomical platform (Biosense-Webster, Diamond Bar, CA, USA). Areas presenting high voltage >0.5 mV, coded with magenta colour, represent conducting tissue. Areas presenting low voltage <0.01 mV, coded with red colour, represent non-conducting tissue. Therefore, all areas within superior vena cava filled with magenta colour represent myocardial sleeves (1) and with red colour smooth muscle parts (2). (**A**). Anteroposterior (AP) view; (**B**). Left anterior oblique (LAO) view; (**C**). Posteroanterior (PA) view; (**D**). Right anterior oblique (RAO) view. See Figure 1 for anatomical details.

The extensive distribution of cardiac muscle fibres in the SVC wall, reaching up to 45 mm from the atrium, creates a large area which is potentially susceptible to the formation of ectopic foci or re-entry circuits. The gradual decrease in the amount of muscle fibres towards the periphery may lead to electrical heterogeneity, which also promotes arrhythmias [17] (Figure 4).

## 6. Electrophysiological Characteristics of the SVC and Venoatrial Junction

Despite its clinical significance in arrhythmogenesis, the detailed electrophysiological properties of the SVC and venoatrial junction (VAJ) have received relatively limited research attention compared to other cardiac structures.

The SVC and VAJ demonstrate several distinctive electrophysiological properties that have been characterized through targeted experimental protocols made by Fukumoto, Kotaro et al. [22]. During sinus rhythm, the conduction time from the right atrium to SVC has been measured at approximately 24 ± 14.6 ms. Intracaval conduction delay and decremental conduction properties via the VAJ can be demonstrated using various pacing maneuvers from both the right atrial appendage (RAA) and SVC. Burst pacing from the RAA with gradually shortened cycle lengths reveals various degrees of conduction block over the VAJ, including delayed conduction, Wenckebach-like conduction block, two-to-one conduction block or complete conduction block. Single extrastimulus protocols from the RAA can separate the activation sequence of SVC electrograms from RA potentials, allowing for the clear identification of SVC activation patterns. Notably, the effective refractory period (ERP) of the SVC has been shown to be significantly longer than that of the right atrium, with measurements of 269 ± 56.1 ms and 208 ± 27.7 ms, respectively, during testing with a basic cycle length of 600 ms.

Pacing from the SVC can demonstrate conduction delay over the VAJ, intracaval conduction delay, intracaval sequence changes and echo beats between the SVC and right atrium. The most prominent decremental conduction property across the VAJ is typically induced by single extrastimulus from the RAA rather than burst pacing or SVC stimulation protocols. When using single extrastimulus from the RAA with a basic cycle length of 600 ms, the conduction time from the RA to SVC can be prolonged by 81 ± 49.7 ms, while similar protocols from the SVC prolong conduction by 61 ± 58.7 ms. These findings suggest asymmetric conduction properties across the VAJ that may influence arrhythmia susceptibility [22].

A significant finding from an investigation by Lee was that the ERP of SVC was consistently longer than that of the high right atrium (HRA) [24]. This electrophysiological disparity may partially explain why triggering foci for atrial fibrillation are less commonly observed in SVC compared to pulmonary veins, which conversely exhibit shorter ERPs than the left atrium [25].

Lee and co-workers [24] also demonstrated that electrical remodelling, represented by shortening of ERP, not only occurs in atrial tissue, but also in SVC following short-term moderate-rate pacing from either the HRA or SVC directly. This electrical remodelling was observed after just 5 min of pacing at a cycle length of 400 ms, indicating that even brief periods of tachycardia can induce significant electrophysiological changes in SVC myocardial sleeves. Similar conclusions and effects of remodelling induced by rapid stimulation were reached by the Lee research group [26], which studied dogs.

The researchers noted that such short-term subclinical atrial tachyarrhythmias, which may be clinically silent, could potentially contribute to electrical remodelling in both atrial and thoracic vein tissues. These findings suggest that the tachycardia-induced electrical remodelling of the SVC may significantly contribute to the arrhythmogenic substrate in AF. This observation also has important implications for understanding the progression from paroxysmal to chronic atrial fibrillation.

The effect of drugs on remodelling and a possible change in arrhythmia induction was also studied [24]. One interesting factor was the potential protective effects of calcium channel blockade on electrical remodelling in SVC. Despite the theoretical basis for calcium channel blockers to prevent electrical remodelling through the reduction of calcium overload, intravenous verapamil at clinically therapeutic doses failed to prevent the electrical remodelling of SVC induced by short-term rapid pacing [24,25,26].

## 7. Arrhythmogenic Mechanisms Associated with the SVC

Congenital variants of the SVC can have significant implications for cardiac electrophysiology and arrhythmogenesis. Persistent left SVC is the most common congenital thoracic venous anomaly with a prevalence of less than 0.5% in the general population and up to 10% in patients with congenital heart disease [27]. This may create substrates for arrhythmias, especially with an absent right SVC [15]. When a persistent left SVC connects to the right atrium via a dilated coronary sinus, it can cause stretching of the arteriovenous node and the bundle of His, potentially leading to cardiac arrhythmias, such as atrial and ventricular fibrillation [28].

Multiple factors contribute to the arrhythmogenic potential of the SVC, making it an important non-PV trigger site for atrial fibrillation. The electrophysiological characteristics of the SVC and VAJ, particularly the decremental conduction properties and potential for re-entry, may directly contribute to the onset and maintenance of AF [22]. Previous research with the use of multielectrode basket has shown re-entry over the PV–LA junction in AF patients, suggesting that its decremental conduction properties may serve as a potential substrate for the initiation and maintenance of AF. Patients with re-entry demonstrated a significantly shorter ERP of distal PVs (127 ± 60 vs. 199 ± 37 ms, *p* < 0.01), longer conduction heterogeneity (60 ± 43 vs. 30 ± 15 ms, *p* < 0.01) and more pronounced conduction delay from the distal PV to PV–LA junction (110 ± 51 vs. 72 ± 25 ms, *p* < 0.01) than those without re-entry. The presence of ERP heterogeneity and anisotropic conduction properties within the PV and at the PV–LA junction proved crucial in promoting re-entry formation and potentially playing an important role as a substrate for the maintenance of AF [29].

Though focused on pulmonary veins rather than the superior vena cava, these findings offer valuable comparative information about myocardial sleeves and their electrophysiological behaviour.

The observed electrophysiological behaviours of the SVC–VAJ complex bear similarities to those documented in the pulmonary veins and PV–left atrial junction of AF patients, suggesting comparable arrhythmogenic mechanisms [30,31,32].

An important distinction between pulmonary vein and SVC electrophysiology lies in their refractory period characteristics. According to previous studies, the ERP of the pulmonary veins is generally shorter than that of the PV–LA junction, whereas Fukumoto, Kotaro et al. demonstrated that the ERP of the SVC is longer than that of the VAJ [22].

Currently, data on the detailed electrophysiological properties of the superior vena cava and its arrhythmogenicity are limited. Previous studies of SVC myocardial sleeves in animal models, and a number on the cellular level, offer interesting observations.

The arrhythmogenic potential of SVC myocardial sleeves stems from multiple cellular mechanisms that promote abnormal electrical activity. Autonomic influences, including both sympathetic and parasympathetic stimulation, can significantly change the arrhythmogenic propensity of SVC sleeves. A consequence can be the promotion of spontaneous automaticity and triggered activity, thereby generating extrasystolic activity capable of initiating atrial arrhythmias [33]. Similar properties have been found in studies of pulmonary veins, well known for their arrhythmogenicity [34,35]. The presence of pacemaker activity cells [36] and characteristic phase 4 depolarization in SVC sleeve preparations indicate an inherent potential for enhanced automaticity, which serves as an important mechanism for the genesis of atrial arrhythmias originating from the SVC [33]. Experimental evidence demonstrates that SVC sleeves can develop late phase 3 early afterdepolarizations (EADs)- and delayed afterdepolarizations (DADs)-induced triggered activity following exposure to acetylcholine, isoproterenol, high calcium or a combination of these factors. This triggered activity represents a critical mechanism by which SVC sleeves initiate atrial arrhythmias. Isoproterenol, a sympathetic agonist, frequently induces an increase in automaticity in SVC sleeves by enhancing the slope of phase 4 depolarization, leading to spontaneous electrical activity [33].

The cellular mechanisms underlying phase 4 depolarization in SVC may involve the Lakatta “calcium clock” [37] hypothesis described for sinus node cells, suggesting that spontaneous calcium releases contribute to the accentuation of phase 4 depolarization.

Another study on dogs showed that myocardial extensions are anatomically positioned in close proximity to the SVC–aorta ganglionated plexus (SVC–Ao–GP), a fat pad containing autonomic neural elements located near the junction of the SVC, right pulmonary artery and aorta. Studies have shown that the SVC–Ao–GP influences the electrical properties of the SVC sleeves preferentially, as evidenced by the significant shortening of the effective refractory period and increased vulnerability to arrhythmias upon stimulation of this ganglionated plexus [38]. This selective autonomic modulation creates an electrophysiological substrate conducive to the initiation of rapid firing originating from the SVC, potentially triggering atrial fibrillation. When hyperactivity of the SVC–Ao–GP is induced, either through electrical stimulation or direct acetylcholine administration, rapid firing from the SVC can be consistently provoked, suggesting a mechanistic relationship between autonomic neural elements and arrhythmogenic activity in this region.

Another study by Lu et al. [39] investigated the role of the intrinsic cardiac autonomic nervous system (ICANS) in initiating rapid focal firing and AF through systematic ablation of GP in a canine model. Ablation of right-sided GP (anterior right GP [ARGP] and inferior right GP [IRGP]) significantly increased the AF threshold at ipsilateral right atrial and PV sites, while left-sided GP ablation (superior left GP [SLGP], inferior left GP [ILGP] and ligament of Marshall [LOM]) elevated thresholds in left-sided structures, highlighting the interconnected neural network governing these regions. This evidence positions the ICANS as a critical regulator of focal arrhythmias in thoracic venous structures, including the SVC, and supports the potential therapeutic value of autonomic modulation in managing SVC-related arrhythmogenesis.

## 8. Electroanatomical Correlations and Clinical Implications

The superior vena cava represents a critical anatomical structure in the pathophysiology of atrial fibrillation, particularly as a source of non-pulmonary vein ectopic foci. Currently, thanks to the accuracy of modern 3D mapping systems, it is possible to accurately assess the size and electrophysiological properties, including an analysis of potential and activation maps, of the muscular structure within the SVC.

One of the most recent studies by Yamagishi et al. [5] investigated the relationship between ERP characteristics of the SVC and the inducibility of atrial fibrillation following pulmonary vein isolation procedures. The myocardial sleeve length was measured from the SVC–right atrial junction to the top of the myocardial sleeve, with both long and short diameters of the SVC assessed at the junction level. Patients with inducible atrial fibrillation demonstrated significantly longer SVC diameters (27.4 ± 4.3 vs. 22.9 ± 4.6 mm, *p* = 0.03) compared to non-inducible patients, suggesting that larger SVC dimensions contribute to enhanced arrhythmogenic substrate. The electrophysiological characterization of SVC myocardial sleeves revealed distinct patterns in effective refractory period distribution and electrical properties. Measurements were systematically performed at three anatomical positions: anterior, septal and posterior aspects. The overall mean SVC-ERP across all patients was 273.8 ± 43.4 ms, with regional variations showing 280.0 ± 49.0 ms anteriorly, 269.2 ± 46.0 ms septally and 272.1 ± 45.0 ms posteriorly. Patients with inducible atrial fibrillation demonstrated significantly shorter average SVC-ERP (236.0 ± 25.2 vs. 294.8 ± 36.8 ms, *p* < 0.001) compared to non-inducible patients. Importantly, the dispersion of SVC-ERP, defined as the difference between longest and shortest refractory periods, showed no significant difference between groups (30.0 ± 25.4 vs. 33.3 ± 20.1 ms, *p* = 0.56), indicating that absolute refractory period values rather than heterogeneity determine arrhythmogenic potential.

A comprehensive study by Nyuta et al. investigated the properties of SVC in 427 consecutive patients with non-valvular atrial fibrillation undergoing radiofrequency catheter ablation. The study revealed that the length of the myocardial sleeve from the top of the sinus node to the top of the myocardial sleeve in the SVC (L-SVC) was significantly longer in patients with SVC firing compared to those without (41.9 ± 12.5 mm vs. 24.9 ± 12.3 mm; *p* < 0.001). Additionally, both the longer diameter (20.1 ± 2.7 mm vs. 18.6 ± 4.3 mm; *p* < 0.001) and shorter diameter (18.6 ± 2.8 mm vs. 17.3 ± 5.0 mm; *p* = 0.004) of the SVC were significantly greater in patients exhibiting SVC firing [40]. The local activation time (LAT) of the SVC was significantly longer in patients with SVC firing compared to those without (81.5 ± 22.7 ms vs. 75.8 ± 28.2 ms; *p* = 0.035). This prolonged activation time likely reflects the increased atrial muscle mass within longer and wider myocardial sleeves, which may contribute to enhanced arrhythmogenicity.

Another study compared data from three-dimensional maps of the superior vena cava in patients with AF and compared them to a control group without a history of AF. The study by Chen et al. aimed to provide an anatomical basis for safer SVC isolation procedures by characterizing myocardial sleeve dimensions and sinoatrial node positioning. The mean length of the SVC myocardial sleeve measured by electroanatomic mapping was 39.8 ± 9.5 mm in patients with atrial fibrillation. Notably, no significant difference was observed in myocardial sleeve length between patients with AF and non-AF controls (39.8 ± 9.5 mm vs. 35.7 ± 8.5 mm, *p* = 0.07). Similarly, sleeve length did not differ between paroxysmal AF and persistent AF patients (39.4 ± 9.0 mm vs. 40.5 ± 10.7 mm, *p* = 0.51). However, significant gender-related differences were identified, with male patients demonstrating longer myocardial sleeves compared to females [41]. The research demonstrated that 52.9% of patients had sinoatrial node earliest activation located above the RA–SVC junction, within the SVC itself. This anatomical variability emphasizes the importance of accurate electroanatomical mapping before SVC isolation to prevent inadvertent sinoatrial node injury, which occurs in 2–4.5% of cases [41].

Kiedrowicz R et al. have proven in their study that despite effective isolation of the pulmonary vein ostia, atrial fibrillation can be triggered by the conduction of firing activity from the right superior pulmonary vein to the superior vena cava. During sinus rhythm, electrical conduction can propagate from the SVC to the right upper pulmonary vein (RUPV), while during arrhythmogenic episodes, reverse conduction can occur from the RUPV to the SVC [42]. This bidirectional electrical communication challenges traditional approaches to pulmonary vein isolation. The superior vena cava demonstrates complex electrophysiological interactions with adjacent pulmonary veins, particularly the RUPV. Anatomical proximity plays a crucial role in these interactions, with the anterior part of the RUPV positioned in a close relationship to the posterior aspect of the SVC, creating potential conduction pathways between these structures. These anatomical connections allow electrical signals to bypass standard pulmonary vein isolation procedures, potentially explaining cases of procedural failure.

## 9. Superior Vena Cava Isolation (SVCI)

**Figure 5:**
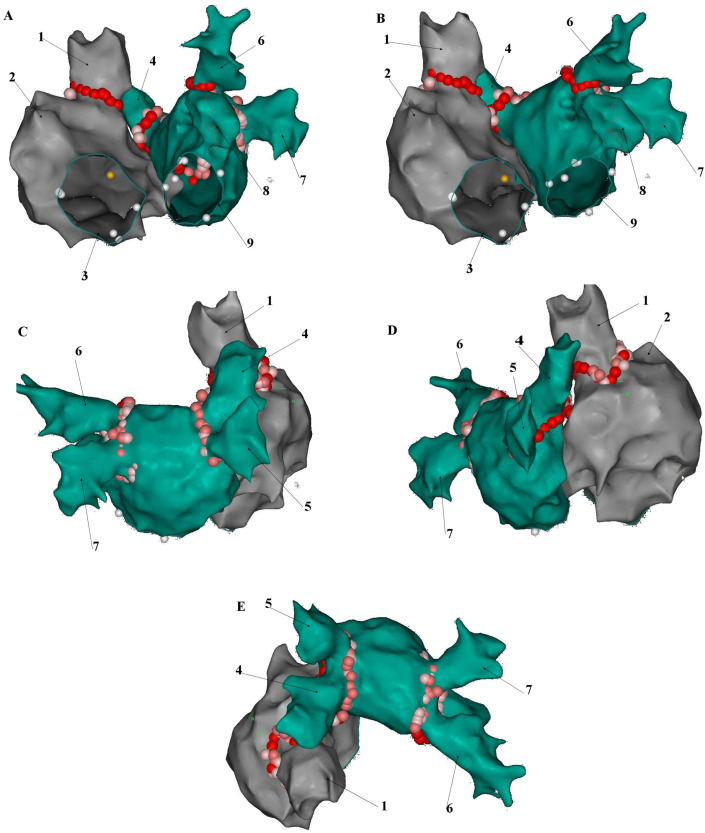
Point-by-point, circumferential, low energy radio-frequency superior vena cava isolation added on the top of pulmonary isolation with a Smarttouch SF catheter using CARTO^®^ 3 platform (Biosense-Webster, Diamond Bar, CA, USA). (**A**). Left anterior oblique (LAO) view; (**B**). Left lateral (LL) view; (**C**). Posteroanterior (PA) view; (**D**). Right Lateral (RL) view; (**E**). Superior view. 1—Superior vena cava, 2—Right atrial appendage, 3—Tricuspid valve annulus, 4—Right upper pulmonary vein, 5—Right lower pulmonary vein, 6—Left upper pulmonary vein, 7—Left lower pulmonary vein, 8—Left atrial appendage, 9—Mitral valve annulus.

SVCI may become an increasingly common adjunctive strategy in the catheter ablation of atrial fibrillation. The vast majority of relevant clinical data are based on radio frequency (RF) point-by-point ablation combined with 3D mapping systems (Figure 5). However, other ablation strategies have been successfully applied (Table 2).

## 10. RF Ablation

The most recent meta-analysis by Mariani et al. (2024) analyzed four randomized controlled trials encompassing 600 patients and demonstrated that empirical SVCI combined with pulmonary vein isolation (PVI) significantly reduces atrial fibrillation recurrence in paroxysmal AF patients compared to PVI alone (11.7% vs. 19.9%, RR 0.54 [0.32–0.92], *p* = 0.02) [43]. Repeat ablation studies demonstrate particular benefits from empirical SVC isolation. In patients undergoing redo procedures after failed PVI, empirical SVCI represents an independent predictor of freedom from recurrence (95% CI: 1.64–32.8, *p* = 0.009). This suggests that SVC isolation may be particularly valuable in PVI non-responders [44]. With the development of new technologies, there are new emerging reports about their additional usefulness in the field of superior vena cava isolation.

### 10.1. SVC Isolation Approaches

Several studies have described circumferential (“ring”) SVC isolation and segmental (non-circumferential) approaches. Oguri N. et al. randomized 62 patients to segmental SVCI guided by omnipolar conduction-block mapping, versus conventional circumferential ablation. Segmental SVCI required fewer RF applications (13.6 ± 6.0 vs. 19.8 ± 10.9, *p* = 0.046) and shorter procedure times (9.6 ± 6.8 vs. 14.2 ± 7.5 min, *p* = 0.032), with equivalent acute isolation rates and safety profiles [45].

A study by M. Haghjoo demonstrated that a segmental strategy demonstrated in 59 patients that high-density mapping of conduction-block lines at the RA–SVC junction enabled the delivery of RF lesions only across gap areas achieving 100% acute isolation without phrenic nerve or sinus node injury or late SVC stenosis [46]. Chen C presented “The C-Shaped Approach”, his study involved 12 patients undergoing partial, non-circumferential lesion sets that selectively targeted the arrhythmogenic sleeve while sparing the lateral segment, achieving 100% bidirectional block with no acute complications [47].

Together, these data indicate that segmental SVCI—whether guided by conduction-block mapping, omnipolar technology, or anatomically tailored C-shaped lesion sets—achieves comparable acute isolation success to circumferential SVCI, while reducing RF burden, shortening procedure time and sparing non-target tissue, thereby minimizing phrenic nerve injury, sinus node damage and late stenosis risk.

### 10.2. Very High-Power, Short Duration (vHPSD) RF Ablation

In a recent study by Fujimoto et al. vHPSD (90 W for 3 s) RF ablation was applied to SVC isolation with excellent procedural efficiency. In a recent cohort of 47 patients, 27 were treated with vHPSD and 20 underwent conventional low-power RF (20–40 W) isolation. The group with vHPSD achieved 100% first-pass SVC isolation, compared to 85% with conventional low-power delivery (*p* = 0.04), while reducing mean ablation points and total RF time (9 ± 4 min vs. 17 ± 5 min, *p* < 0.01) [48].

### 10.3. Pulsed-Field Ablation (PFA)

Ollitrault et al. suggested that PFA has emerged as a highly promising modality for superior vena cava isolation, offering rapid tissue-selective lesion formation and achieving 100% acute isolation success in a recent study [49]. However, all published PFA studies to date focus on acute procedural outcomes, and long-term efficacy and durability data remain limited. Until larger, multicenter trials with extended follow-up become available, the long-term benefits of PFA in SVC isolation cannot be fully established [49,50].

### 10.4. Cryoballoon Ablation (CBA)

There are a couple of reports concerning CBA-based SVC isolation, that proved its feasibility and safety. However, a randomized CAVAC-AF trial found that adding cryoballoon SVC isolation to PVI did not improve arrhythmia outcomes but did increase transient complications. The study included 98 patients with paroxysmal or non-long-standing persistent AF. Patients were randomized to PVI alone or PVI plus empirical SVC isolation. After 12 months, freedom from atrial tachyarrhythmia was 62.9% in the PVI+SVCI group versus 72% in the PVI-only group (*p* = 0.41), indicating no significant advantage for SVC isolation. In conclusion, the results do not support routine SVC isolation by cryoballoon in first-time ablation patients with paroxysmal or non-long-standing persistent AF [51].

### 10.5. Complications Associated with Superior Vena Cava Isolation

Based on the meta-analysis of 600 patients across four randomized controlled trials, RF SVC isolation demonstrates a favourable safety profile with low overall complication rates. The pooled analysis revealed no significant difference in complication rates between SVCI combined with PVI versus PVI alone (OR 1.06, 95% CI 0.33–3.44, *p* = 0.92) [43].

### 10.6. Phrenic Nerve Injury (PNI)

The most notable complication reported seemed to be PNI, occurring specifically in the study by Da Costa et al., where two cases were documented. The overall incidence of PNI during SVCI remains low at approximately 0–5% and is typically transient in nature [43]. This complication can be effectively prevented by several approaches. (a) High-output pacing: Pre-ablation identification of phrenic nerve capture using high-output pacing (30 mA) represents the primary preventive strategy [52]. (b) Intracardiac echocardiography (ICE) visualization: The right phrenic nerve can be directly visualized using ICE during atrial fibrillation ablation, thereby preventing injury during radiofrequency energy delivery [53]. (c) HPSD technique: Yamaji et al. demonstrated that HPSD radiofrequency energy application (50 W for 7 s) applied only to SVC points where pacing stimulates the phrenic nerve never resulted in PNI [54]. This approach may represent optimal prevention due to the creation of shallower and wider lesions compared to standard radiofrequency ablation.

### 10.7. Sinus Node Injury

No cases of sinus node injury were reported in this meta-analysis, despite a previous study wherein 354 patients underwent ablation in or around the SVC and the investigating group documented this complication in 1.1% of cases [55]. Modern electroanatomical-mapping-guided SVCI enables precise localization of the sinus node, allowing successful ablation without sinus node damage [12].

### 10.8. SVC Stenosis

SVC stenosis is another possible complication that must be considered when considering vein isolation. Although a large meta-analysis showed no data of its presence, based on a case described by Kühne, Michael et al., severe stenosis of the SVC after RF ablation to obtain SVC isolation using an irrigated-tip catheter may occur [56]. In another study of 13 patients, local and circumferential swelling was observed during RF application within the SVC–RA junction, causing a progressive reduction in the diameter of the SVC–RA junction by 24% [57].

In the context of very high-power short-duration ablation for SVC isolation, no increase in major complications was observed, and phrenic nerve injury remained transient and self-limited. These findings suggest that vHPSD may optimize both safety and efficiency in SVC isolation [45].

The tissue-selective properties of PFA minimize collateral damage to critical structures, with only 4.7% transient sinus node dysfunction and complete resolution of all complications by procedure end [49].

Complications were notably higher with cryoballoon SVC isolation. Phrenic nerve paralysis occurred in 20.8% of the PVI+SVC group versus 6% in the PVI-only group (*p* = 0.003), and transient sinus node injury was seen in 18.8% versus 0% (*p* = 0.001). Most complications resolved within 24 h, though one case of phrenic paralysis persisted for three months.

## 11. Conclusions

The superior vena cava has emerged as a critical anatomical structure in the pathophysiology of atrial fibrillation, representing one of the most common sources of non-pulmonary vein foci responsible for AF initiation and maintenance. While pulmonary vein isolation remains the cornerstone of AF ablation, the recognition of SVC’s arrhythmogenic potential has recently led to increased interest in its electrophysiological and electroanatomical characteristics and in SVC isolation as an adjunctive therapeutic strategy. Understanding the properties of the SVC is essential for optimizing ablation outcomes while minimizing procedural complications.

## 12. Future Directions

The superior vena cava and its arrhythmogenicity responsible for AF have received relatively limited research attention. Therefore, we strongly recommend that future research should focus on: (a) The establishment of the actual number of patients exhibiting SVC-firing-induced AF. (b) Detailed electrophysiological properties of SVC and its arrhythmogenicity. (c) Electroanatomical characteristics of the SVC–RUPV junction and finally, (d) to establish whether SVC isolation on the top of PVI could help to improve AF ablation outcomes.

## Figures and Tables

**Table 1:** The prevalence of SVC serving as the non-PV triggers across the studies.

Author (Year)	Patients Included	Global Number of Non-PV Triggers	SVC Arrhythmogenic Foci in Non-PV Triggers Cohort	SVC Arrhythmogenic Foci in Overall Cohort
Lin et al. (2003) [8]	240 (paroxysmal AF)	73/240 (30.4%)	27/73 (37.0%)	27/240 (11.3%)
Takigawa et al. (2017) [9]	865 (paroxysmal AF)	125/865 (14.5%)	68/125 (45.6%)	68/865 (7.9%)
Kawai et al. (2022) [10]	1020 (paroxysmal + non-paroxysmal AF)	126/1020 (12.4%)	29/126 (23%)	29/1020 (2.8%)
Miyata et al. (2024) [11]	1089 patients/1160 procedures (paroxysmal + non-paroxysmal AF)	160/1160 (13.8%)	64/160 (40%)	64/1160 (5.5%)
Dong et al. (2024) [12]	130 (paroxysmal AF)	43/130 (33.1%)	30/43 (69.8%)	30/130 (23%)

**Table 2:** The advantages and disadvantages of different SVCI techniques.

Ablation Technique	Clinical Data						
	Procedural Time	Radiation Exposure	Acute Success Rate	Long-Term Success Rate	Phrenic Nerve Palsy	Sinus Node Dysfunction	SVC Stenosis
Point-by-point low-power RF ablation integrated with 3d mapping system	+++	+	+	+++	+++	+++	+
Point-by-point very high-power short duration RF ablation integrated with 3d mapping system	++	+	++	++	++	++	+
Single Shot Pulsed Field Ablation under fluoroscopy	+	+++	+++	data pending	+	+	+
Cryoballoon ablation	+	++	+	+	++++	++++	+

Procedural time: + short; ++ relatively short; +++ long; Other features: + low; ++ moderate; +++ high; ++++ very high.

## Abbreviations

The following abbreviations are used in this manuscript:

AF	atrial fibrillation
PV	pulmonary vein
SVC	superior vena cava
PVI	pulmonary vein isolation
LA	left atrium
RA	right atrium
VAJ	venoatrial junction
RAA	right atrial appendage
HRA	high right atrium
ERP	effective refractory period
EADs	early afterdepolarizations
DADs	delayed afterdepolarizations
SVC-Ao-GP	SVC-aorta ganglionated plexus
ICANS	intrinsic cardiac autonomic nervous system
ARGP	anterior right GP
IRGP	inferior right GP
SLGP	superior left GP
ILGP	inferior left GP
LOM	ligament of Marshall
L-SVC	myocardial sleeve in the SVC
LAT	local activation time
RUPV	right upper pulmonary vein
SVCI	superior vena cava isolation
RF	radio frequency
vHPSD	very high-power, short duration
PFA	pulsed-field ablation
CBA	cryoballoon ablation
PNI	phrenic nerve injury
